# Fast and Accurate Light Field View Synthesis by Optimizing Input View Selection

**DOI:** 10.3390/mi12050557

**Published:** 2021-05-13

**Authors:** Xingzheng Wang, Yongqiang Zan, Senlin You, Yuanlong Deng, Lihua Li

**Affiliations:** 1 College of Mechatronics and Control Engineering, Shenzhen University, Shenzhen 518060, China; xingzheng.wang@szu.edu.cn (X.W.); zanyongqiang@163.com (Y.Z.); senlinyou@foxmail.com (S.Y.); dengyl@szu.edu.cn (Y.D.); 2 Sino-German College of Intelligent Manufacturing, Shenzhen Technology University, Shenzhen 518118, China

**Keywords:** light field, depth estimation, view synthesis, convolutional neural network

## Abstract

There is a trade-off between spatial resolution and angular resolution limits in light field applications; various targeted algorithms have been proposed to enhance angular resolution while ensuring high spatial resolution simultaneously, which is also called view synthesis. Among them, depth estimation-based methods can use only four corner views to reconstruct a novel view at an arbitrary location. However, depth estimation is a time-consuming process, and the quality of the reconstructed novel view is not only related to the number of the input views, but also the location of the input views. In this paper, we explore the relationship between different input view selections with the angular super-resolution reconstruction results. Different numbers and positions of input views are selected to compare the speed of super-resolution reconstruction and the quality of novel views. Experimental results show that the speed of the algorithm decreases with the increase of the input views for each novel view, and the quality of the novel view decreases with the increase of the distance from the input views. After comparison using two input views in the same line to reconstruct the novel views between them, fast and accurate light field view synthesis is achieved.

## 1. Introduction

Light field cameras can record angular and spatial information of a scene simultaneously [1]. The micro-lens array is the core of the light field camera, and it is also the difference between a light field camera and a traditional camera. The fabrication methods of the micro-lens array can be divided into direct and indirect ones. Direct methods include the reflow methods [2,3], ink-jet printing technique [4,5], etc. [6]. The direct methods are simple, but it is difficult to control the accuracy of the micro-lens array. Direct methods include methods based on a micro-electro-mechanical system [7,8] and methods using ultra-precision machining [9,10]. The indirect methods can effectively control the shape accuracy of the micro-lens array.

The angular resolution is the basis of many applications [11,12,13]. For example, it directly affects the accuracy of depth map estimation and then influences the effect of 3D reconstruction. The accuracy of the depth map estimation is proportional to the angular resolution [14].

Limited by the structure of the light field camera and the resolution of the sensor, there is an inevitable trade-off between spatial and angular resolution, i.e., a high spatial resolution will lead to low angular resolution. In many applications, both high spatial and angular resolution are needed to be guaranteed simultaneously. Higher angular resolution refers to more view images and higher spatial resolution stands for more details in each view image. For instance, in a 3D display, enough views and spatial resolution are essential to provide a truly immersive 3D experience [15].

Many scholars have studied how to improve the angular resolution while ensuring the high spatial resolution, which is also called light field view synthesis [16,17,18,19]. Wanner et al. [20] first estimate the depth at the input views and use it to warp the input images to the novel view. With the development of deep learning, learning-based light field angle super-resolution reconstruction methods have been proposed. The commonly used methods can be divided into two categories.

The first category does not need depth estimation. For the first time, Yoon et al. [17] applied a convolution neural network (CNN) to light field angular super-resolution and improved the quality of novel views. However, their method can only synthesize the view between two adjacent views. M. Shahzeb Khan Gul et al. [14] designed a method to work on raw light field data to synthesize novel views rather than on view images. Their method could only enhance the angular resolution by a factor of two. Meng et al. [21] formulated light field super-resolution as tensor restoration and developed a learning framework based on two-stage restoration with four-dimensional convolution. However, the methods above can only synthesize the view between two adjacent views. In other words, the methods above could only enhance the angular resolution by a factor of two. Wu et al. [22] model the problem as a learning-based angular detail restoration on EPI. Nevertheless, the method relying on EPI structures works well only if the baseline is small [18].

The second category divides view synthesis into two parts: depth estimation and view synthesis. These approaches use deep-learning methods to build the relationship between input views and novel views. Kalantari et al. [16] present a learning-based method for light field view synthesis. They used only four corner views as inputs to reconstruct views at arbitrary locations. Based on Kalantari’s work, Deng Wu et al. [23] reconstructed a novel view using global and local multi-views, avoiding the loss of objects and yielding a better reconstruction effect. Li Kun et al. [24] synthesized high-quality novel views by CNN with Resnet blocks. Shi et al. [18] used a fusion module to merge pixel and feature-based view synthesis for light fields, improving the quality of synthesized novel views. They also designed an interpolation method to solve the occlusion problem in view synthesis. The advantage of these methods is that they can rebuild novel views at an arbitrary location by using only four corner views as input. In the experiment, we found that the algorithm is slow because each novel view requires four input views to participate in the calculation. In practical application, the speed of the algorithm and the accuracy of novel views are both important. Taking light field compression as an example [25], light field records multiple views and contains a lot of information, which is not conducive to the storage of light field data. Therefore, we can record a small number of views for storage and synthesize a large number of views when needed. The faster the synthesis of the novel view, the higher the efficiency of reconstruction. At the same time, we hope to reconstruct a novel view image that is closer to the ground truth image.

Therefore, in this paper, we explore different input view selections for depth estimation based light field angular super-resolution methods. We change the number and position of input views to compare the speed of the reconstruction and the quality of the novel views. Experimental results show that compared with using only four input views, using two views in the same line as the input to reconstruct the intermediate novel views speeds up the algorithm by nearly 40% as well as improving the novel view quality.

## 2. Methods

### 2.1. View Synthesis Method

Among many depth-estimation-based light field angular super-resolution reconstruction methods, we choose Kalantari’s method to experiment with. Kalantari is the first to apply the deep learning method to light field super-resolution reconstruction based on depth estimation. His algorithm framework includes two parts: depth estimation and color estimation, which can reconstruct the view of any position with only four corner views. As he uses a learning-based approach to learn the complex relationship between the novel views and the input views, the quality of the novel views far exceeds the previous algorithms. At the same time, he created the light field data set for network training. 

Other depth estimation based light field angular super-resolution reconstruction methods [18,23] also show that different input views have an impact on the reconstruction of novel views, and in this paper, we undertake a specific investigation.

**Figure 1:**
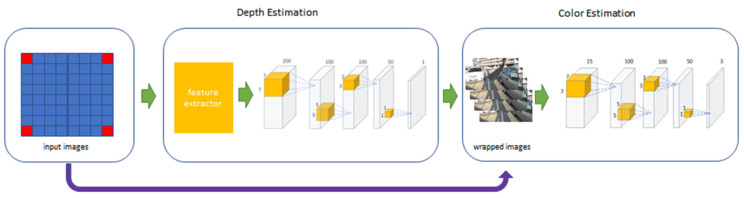
The framework of Kalantari’s method, input views are shown in red.

The framework of Kalantari’s method is shown in Figure 1. The light field includes 8×8 views and four corner views are selected as the input. We changed the number of input views for each novel view reconstruction from 2 to 5, retrained the network, and tested the results of novel view reconstruction.

### 2.2. Set-Up

We implemented our experiment in MATLAB R2017a and used MatConvNet [26] for the network. The experiment was run on CPU Intel i5-9400 2.90GHz 16GB RAM. We recorded the running time on a GPU of type NVidia GeForce RTX 2060Super.

The data set was provided by Kalantari and includes 100 training images and 30 test images.

### 2.3. Evaluation Principle

Our goal is to reconstruct the novel view $L_{q}\;$by using the input views$\;L_{p}$, which can be expressed as:

(1) $$L_{q}=f\left(L_{p1},\ldots ,L_{pn}\right)$$

where n in Equation (1) represents the number of input views. We compared the speed of novel view reconstruction T and the quality of novel views Q. The factor that affects the reconstruction speed T is the number of input views for each novel view n. The factors affecting the quality of the novel views Q include the number of input views n and position of the input view p. These can be expressed as:

(2) T=g(n)

(3) Q=h(n,p)

We use the time needed to synthesize a novel view as the evaluation principle of algorithm speed T. We use peak signal-to-noise ratio (PSNR) and structural similarity (SSIM) to evaluate the quality of the novel views Q. At the same time, we compare the visual effects of the novel views.

The time required for Kalantari’s method to synthesize a novel view is $T_{0}$, and the quality of the novel view is $Q_{0}$. If T <$\;T_{0}$, we think the algorithm speed is improved. If Q >$\;Q_{0}$, we think the quality of the novel views is improved. We hope to improve the speed of the algorithm and the quality of the novel views by optimizing the input view selection.

### 2.4. Input View Selections

Light field cameras can record multi-views. There is a horizontal disparity between the views in the same line and a vertical disparity between the views in different lines. Kalantari used four corner views as input for each novel view reconstruction, as can be seen in Figure 1. This is because the four corner views contain sufficient information to reconstruct other views.

First, based on four input views, the number of input views can be increased, but the algorithm speed will be reduced. Then, we consider reducing the number of input views for each novel view reconstruction.

**Figure 2:**
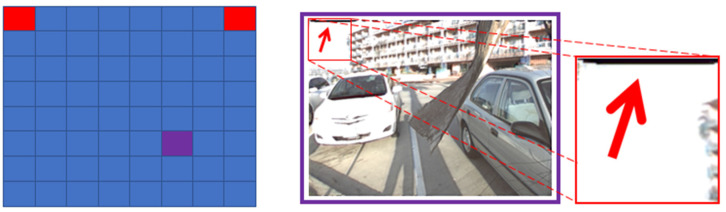
Novel view (6,6) reconstructed by using views (1,1) and (1,8) as input.

When n=2, from Figure 2, we can see that black edges appear when reconstructing novel views (6,6; the view of the sixth row and the sixth column) using two input views (1,1) and (1,8). This is because there is only a horizontal disparity between the views in the same line, while there is a vertical disparity between the views in different lines. Therefore, two input views in the same line can provide enough information for the view between them but cannot provide enough information for the view in different lines.

**Figure 3:**
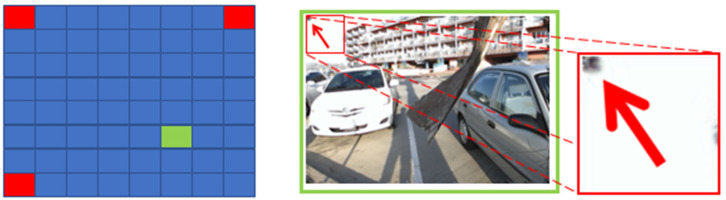
Novel view (6,6) reconstructed by using views (1,1), (1,8), and (8,1) as input.

When n=3, from Figure 3, we can see that only three corner views as input cannot provide enough information for all novel views. The reason is similar to that when n=2.

**Figure 4:**
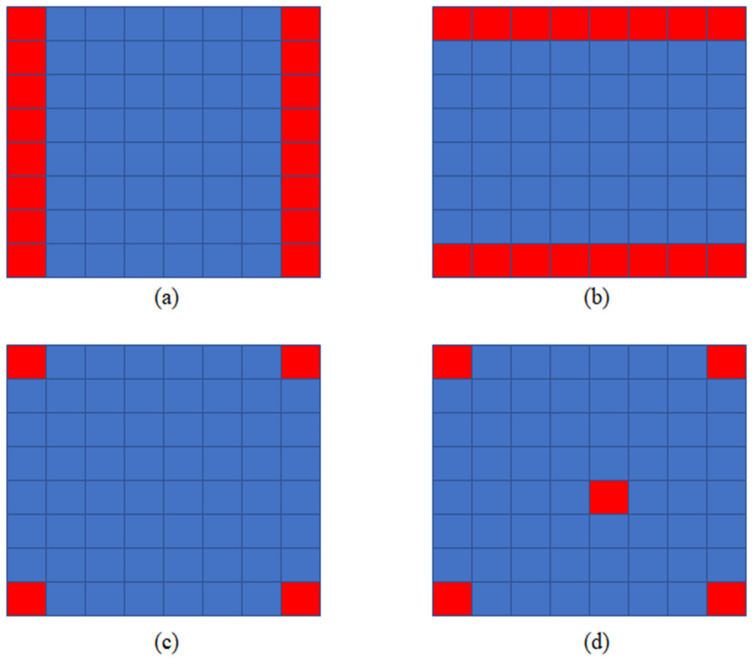
Four input view selections, input views are shown in red. (**a**) Each novel view is synthesized by using two views in the same line as input; (**b**) each novel view is synthesized by using two views in the same column as input; (**c**) each novel view is synthesized by using four corner views as input; and (**d**) each novel view is synthesized by using five views as input.

After the above analysis, we determined four input view selections, which can be seen in Figure 4. For n=2, we use two views in the same line (column) as input to restore the intermediate views, as shown in Figure 4a,b. The method of Kalantari is shown in Figure 4c, four corner views are selected as input. For n=5, we choose four corner views and a central view as input. In the experiment, we found that the further away from the input view, the worse the quality of the new view. Therefore, we choose the center view because it is in the center of four corner views.

In this article, we use Selection (a), Selection (b), Selection (c), and Selection (d) to describe the four input view selections in Figure 4. We studied the speed of the algorithm T when the number of input view n is in the range of 2 to 5. We studied the novel view quality Q using the four input view selections shown in Figure 4.

## 3. Results

### 3.1. Algorithm Speed

**Figure 5:**
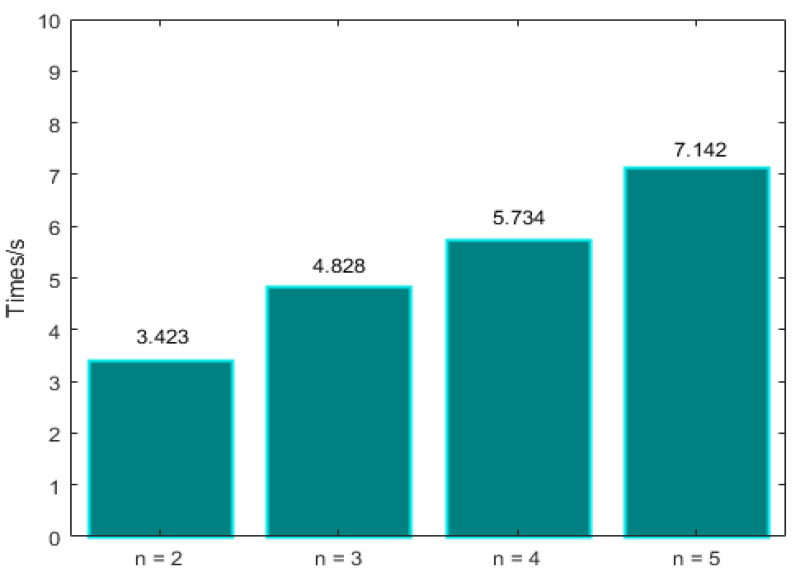
The relationship between algorithm speed and the number of input views.

The process of view synthesis is divided into four steps: prepare depth features, evaluate depth net, prepare color features, and evaluate color net. In the above four steps, the first step takes the most time. Table 1 shows the time spent in each step when the number of input views changes. Figure 5 compares the total time to reconstruct a novel view.

The experimental results show that the speed of the algorithm decreases with the increase of the number of input views for each novel view.

It is important to emphasize that increasing the number of input views for each novel view not only increases the time of angular super-resolution reconstruction but also increases the requirements for hardware. For example, in the process of network training, choosing five input views for each novel view reconstruction requires more memory space.

### 3.2. Novel View Quality

**Figure 6:**
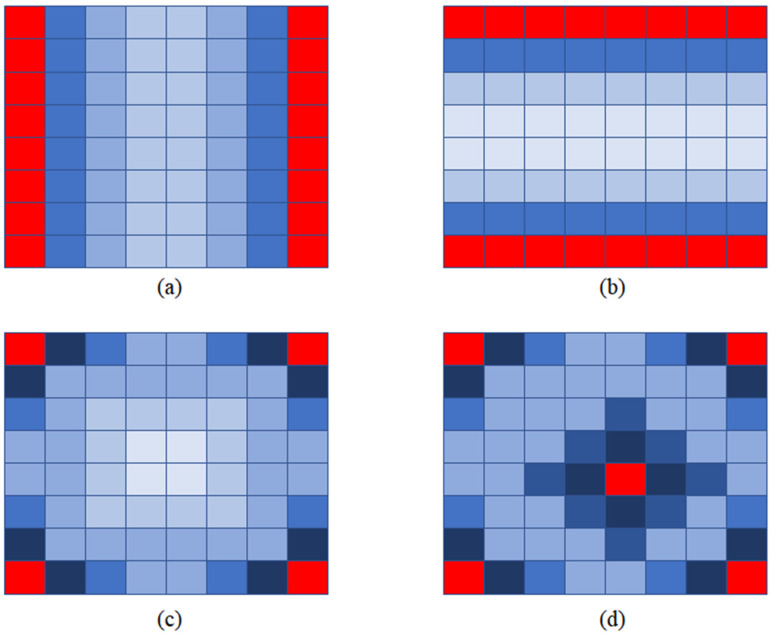
The quality of novel views. Dark blue represents high quality and light blue means low quality. (**a**), (**b**), (**c**) and (**d**) correspond to Selection (a), Selection (b), Selection (c) and Selection (d) respectively.

Five light field images were selected for the test. In the experiment, we found that the quality of the novel view is inversely proportional to the distance from the input views, as can be seen in Figure 6.

**Figure 7:**
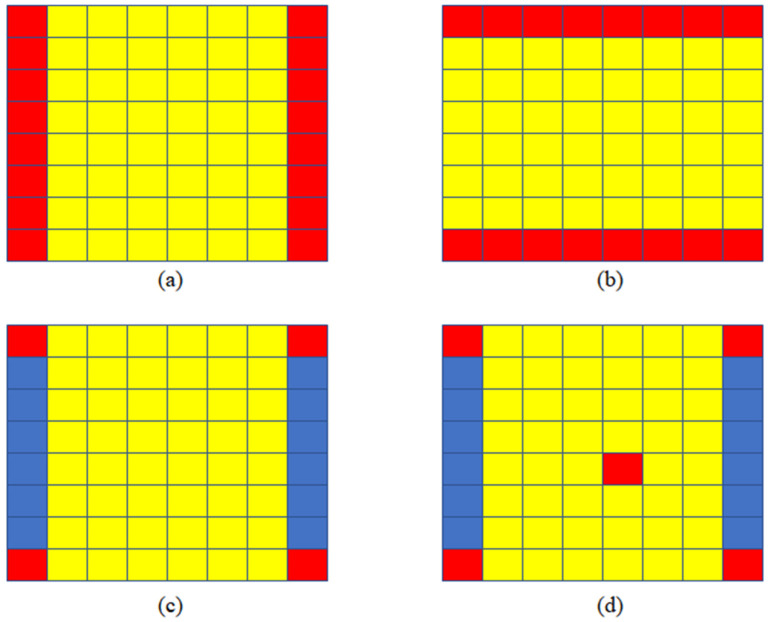
Novel views selected for quality evaluation; the mean value of yellow views is calculated for quality assessment. (**a**), (**b**), (**c**) and (**d**) correspond to Selection (a), Selection (b), Selection (c) and Selection (d) respectively.

To make the comparison fairer, we calculated the mean value of the quality of the yellow views, as shown in Figure 7. Yellow views are selected for quality assessment. and the results are shown in Table 2.

**Figure 8:**
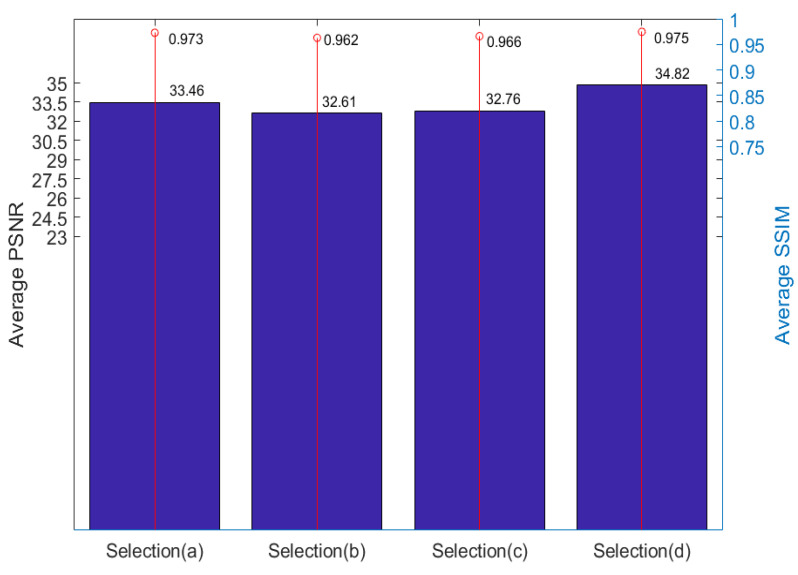
Novel view quality obtained by calculating the mean value of five light field images.

We then calculated the mean value of five light field images, and the result is shown in Figure 8. The result shows that the best effect is achieved by using 5 views as input. Using two views in the same line as input is better than using only four corner views. The reason, as we have said, is that the further away from the input view the lower the quality of the novel view. When using four corner views, the central views are far away from the input views.

**Figure 9:**
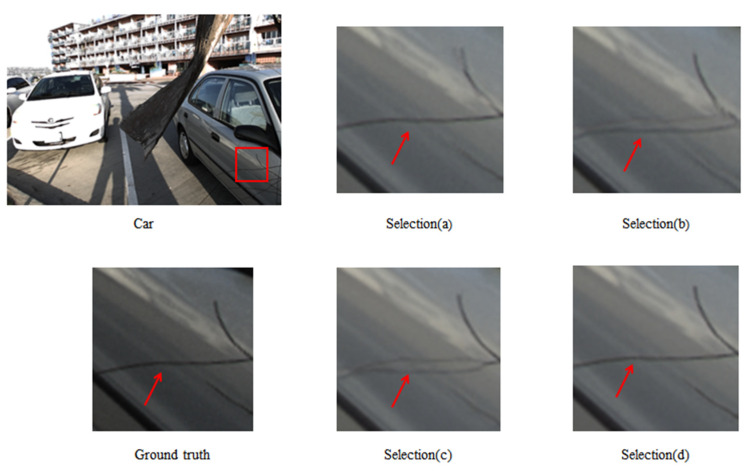
Visual comparison of different input view selections. The novel views synthesized by Selection (a) and Selection (d) are closer to the ground truth. While the novel views synthesized by Selection (b) and Selection (c) have artifacts.

Finally, we did a visual comparison, as shown in Figure 9. The comparison results are consistent with the experimental results. The quality of Selection (a) and Selection (d) reconstruction is high, and the image details are closer to the ground truth. The images reconstructed by using the other two selections have artifacts.

## 4. Discussion

Most of the existing algorithms choose four corner views as input, e.g., Selection (c), but the experimental results show that this selection is not the best in terms of algorithm speed and novel view quality. Increasing the number of input views can improve the quality of new views, such as Selection (d), but it will further reduce the speed of the algorithm. Selection (a) is better than Selection (c) in both the speed of the algorithm and the quality of novel views. Therefore, input view selection can be optimized by using two views in the same line as input.

## 5. Conclusions

Depth estimation based light field angular super-resolution methods can use a small number of input views to reconstruct the novel view at an arbitrary location between them. Most of the existing algorithms use four corner views as inputs, which leads to the slow speed of the algorithm. The speed of the algorithm is related to the number of input views, while the quality of new views is related to the number and location of input views. In this paper, we choose different numbers and positions of input views to compare the speed of the algorithm and the quality of the novel view. Compared with using only four corner views as input, using two input views in the same line can greatly speed up the algorithm while reconstructing high-quality novel views. Therefore, input view selection can be optimized by using two views in the same line as input to achieve fast and accurate light field view synthesis.

## Figures and Tables

**Table 1:** The time taken for each step when the number of input views changes.

Step	*n* = 2	*n* = 3	*n* = 4	*n* = 5
1	3.131	4.504	5.731	6.742
2	0.001	0.001	0.001	0.004
3	0.290	0.322	0.361	0.392
4	0.001	0.001	0.001	0.004

**Table 2:** Novel view quality of different input view selections.

Light Field	Selection (a)		Selection (b)		Selection (c)		Selection (d)	
	PSNR	SSIM	PSNR	SSIM	PSNR	SSIM	PSNR	SSIM
Car	34.03	0.977	32.88	0.971	32.74	0.971	34.26	0.978
Flower	35.23	0.976	34.43	0.972	33.28	0.965	35.34	0.975
Leave	29.99	0.965	30.3	0.941	29.36	0.948	32.54	0.964
Rock	32.96	0.965	32.2	0.954	35.87	0.973	37.14	0.977
Seahorse	35.09	0.981	33.25	0.976	32.56	0.973	34.84	0.979

## Data Availability

Not applicable.
